# Evaluation of Cost-Benefit and Measures During the COVID-19 Pandemic for Incoming Travelers Through Tests in Origin in Spain

**DOI:** 10.3389/fpubh.2022.816406

**Published:** 2022-05-09

**Authors:** Conrado Domínguez, Rubén García, Javier Sánchez, José Pablo Suárez, Carmen Delia Dávila-Quintana

**Affiliations:** ^1^Servicio Canario de la Salud, Consejería de Sanidad, Gobierno de Canarias, Las Palmas, Spain; ^2^Escuela de Doctorado de la Universidad de Las Palmas de Gran Canaria, Las Palmas, Spain; ^3^Centro de Tecnologías de la Imagen (CTIM), Universidad de Las Palmas de Gran Canaria, Las Palmas, Spain; ^4^IUMA Information and Communications System, Universidad de Las Palmas de Gran Canaria, Las Palmas, Spain; ^5^Departmento de Métodos Cuantitativos en Economía y Gestión, Universidad de Las Palmas de Gran Canaria, Las Palmas, Spain

**Keywords:** COVID-19, pandemic, cost-benefit, travelers, RT-PCR test, antigen test

## Abstract

**Background:**

The World Health Organization has promoted preventive measures for reducing the impact of the pandemic. One of these measures was tests in origin for travelers. Testing strategies for COVID-19 facilitate the overall public health response to the pandemic and contributes to minimize the infection among the population COVID-19.

**Goal:**

In this work, we assess the efficiency of diagnostic testing of incoming travelers in the Canary Islands, Spain, during a period of 4 months, with a focus on the economic impact for the regional government. We study the cost-benefit of this measure as well as the potential influence on the number of positive cases in the population.

**Methods:**

We processed the real data in the Canary Islands of pre-flight PCR and antigen tests that were required to the residents when traveling back to the Canaries from anywhere in Spain in a period of 4 months, from 14 December, 2020 to 4 April, 2021. As a result, we calculated the economic impact of doing those tests and compare them with the estimated costs of passengers under the hypothesis of entering the islands without testing. The cost-benefit was obtained for different scenarios, where the incoming passengers generated hospitalization and intensive care unit (ICU) costs directly and *via* transmissions.

**Results:**

The incoming testing funded by the government, if applied during the bad evolution of the pandemic with 1.2 ratio of transmission, clearly saved money to the public health system. In addition to the economic impact of this measure, we estimated the potential influence on the number of positive cases in the population according to different scenarios of the propagation of the pandemic. At the beginning of February 2021, the savings were about €130.551,47, with a 95% confidence interval (CI) of €24.677,94–236.425,00. By the end of April 2021, the savings were above €2,000,000 (€2.284.788,50 on average and 95% CI of €2.092.914,84–2.476.662,16) and the savings increased as the pandemic evolved. At the end of the period, the savings were twice the expenses.

**Conclusions:**

Testing in origin has proved to be a good measure that helped to mitigate COVID-19 spread among regions. Our results confirm that the free PCR or rapid antigen tests produce relevant savings to the public budget. We studied 61.990 reported data during 2020 and 2021 from the travelers from national flights, against 346.449 of total incoming travelers to the Canary Islands in this period. The measure pursued by the Government of the Canary Islands of providing free tests for residents showed a clear benefit for both, limiting the propagation of COVID-19 and reducing the costs of the hospitalizations and ICU admissions. It should be noted that the free testing measure in this period was before starting the vaccination campaigns. As measure of public health in the airports, testing helped to control and make the mobility of travelers secure.

## Introduction

A principal issue questioned by all countries in the very beginning of COVID-19 had been whether keeping borders open might worsen the pandemic or not, and in which terms the borders could be progressively opened without increasing the infections.

At the beginning, testing was done to measure temperature as it may indicate a symptom of COVID-19. A method widely used specially for those spaces with high transit of persons, like airports, supermarkets, etc., was infrared thermal image scanners. However, these methods, or others that measure temperature, were not completely valid to detect the virus. Between 30 and 40% of the people infected with SARS-CoV-2 are asymptomatic and the details of their infection were not clearly known. It had been shown that this group caused a considerable proportion of new cases and transmissions (1). Asymptomatic viral testing strategies for SARS-CoV-2 could facilitate a safe airline travel through reduction of passenger risk of infection and population-level risk from importation.

Fortunately, it has been shown that, when serious hygiene measures are enforced inflight, the transmission rates of SARS-CoV-2 are likely to be exceptionally low, as little as one case per 27.000.000 travelers, even with positive cases aboard (2). It has also been proven that the preventive measures, for example, self-isolating if symptomatic, social distancing, face mask use, hygiene maintenance but primary washing hands are more relevant than quarantining travelers (3). Some other works also corroborate the usefulness of these preventive measures together with short quarantine and post-travel testing to reduce transmission. For example, a detailed simulation study of the USA domestic airline travelers (4) was conducted to estimate a per-day risk of infection with SARS-CoV-2 corresponding to a daily incidence of 150 infections per 100.000 people.

The implementation of testing strategies for COVID-19 facilitates the overall public health response to the pandemic and contributes to minimize the infection among the population. The physical distancing measures have proven to be effective to reduce the incidence of COVID-19 to sporadic cases and control the outbreaks. Implementing travelers testing is a choice for limiting the re-introduction of infections from other regions and becomes increasingly important to prevent additional outbreaks and avoid overwhelming resource-intensive control efforts. Since 3 June, 2020, Singapore has required the visitors from China to take a PCR test no later than 48 h before the departure, with a certificate of their infection-free status needed for entry, and an additional test on arrival (5). A similar policy is in place in Hong Kong; travelers who test positive on arrival are transferred to hospitals (5). Screening and testing procedures, travelers distancing, hygiene measures, and mask use at airports, all during inflight and throughout the entire travel, are proven to be the best solution now. In the work presented in (6), WHO analyzes the requirements and the issues about the testing as a tool for mitigating cross-border transmission of COVID-19. For example, how to increase reliability of testing before or after travel, and evaluating national testing capacities, including laboratory supplies, trained personnel, and personal protective equipment (PPE).

Several countries put in practice the self-quarantine of new arrivals either at home, with family or friends, at hotels or at other temporary accommodations for 14 days (about 2 weeks). Studies show that by day 14, at least 95% of eventually symptomatic cases have become asymptomatic (2). However, the median incubation period for SARS-CoV-2 is about 5 days (2) and assuming that the travelers are equally likely to travel at any point in that period, a 5-day quarantine on arrival seems to be sufficient to allow more than 50% of the infections to develop symptoms and be managed accordingly.

In this work, we evaluated the measures during the COVID-19 pandemic in a period of 4 months, from 14 December, 2020 to 4 April, 2021, for the incoming travelers through testing in the Canary Islands. We analyzed the real data in the Canary Islands of pre-flight PCR and antigen testing, required to the residents when traveling back to the Canaries from any other place in Spain. As a result, we calculated the economic impact of doing those testing and compare them with the estimated the costs of passengers under the hypothesis of entering the islands without testing. Then, a cost-benefit was obtained for different scenarios, where the incoming passengers were derived to hospitalization and intensive care unit (ICU). We showed that a measure of incoming testing funded by the government saved money if applied during the bad evolution of the pandemic (1.2 ratio of transmission). In addition to the economic impact of this measure, we estimated the potential influence on the number of positive cases in the population according to different scenarios of the propagation of the pandemic.

## Materials and Methods

### The PCR and Antigen Test Data

#### Testing Process

A viral test for SARS-CoV-2 was performed on all the travelers coming from any of the territories of Spain who arrived in the Canary Islands between 18 December 18, 2020 and 4 April, 2021. The travelers under 6 years were excluded. The tests could be done in the 72 h prior to arrival in the Canary Islands, or in the 72 h after arrival. It should be noted that the testing in this period was before starting the vaccination campaigns.

The Government of the Canary Islands reached an agreement with a network of laboratories, which serves analytical testing services throughout the territory. Before flying, the travelers should undergo the PCR test, or rapid antigen test, for the detection of Coronavirus in the closest laboratory where they lived.

These tests had been free for the residents. The Canary Islands Health Service [*Servicio Canario de la Salud* (SCS)] arranged with the laboratories to conduct free PCR or rapid antigen tests for residents, as well as a special price for non-residents. In those cases, in which the test could not be done at origin, the airport or port of destination would tell travelers how to proceed. In this case, isolation would have to be kept for the necessary time.

The passenger checkpoints were installed in the six airports in the Canary Islands with arrivals of domestic flights. The travelers who did not present the certificate, or whose result could not be validated, were identified, registered, and isolated until the diagnostic test was carried out, within a maximum period of 72 h after arrival. The residents had been able to access the test through the SCS and non-residents had a discount at the laboratories. However, we did not include the non-resident data in this study.

#### Data Cleaning and Filtering

The company responsible for conducting the tests registered the results in its information system and provided them to the Government of the Canary Islands at the end of the period.

The total number of registers was 61.990, each containing the following information: The day of the test, the gender, and age of the person, the result of the PCR or antigen test, and the name of the province where the laboratory was located. The results were classified as “Positive” or “Negative,” meaning the presence or non-presence of the coronavirus, respectively.

In the first step, we cleaned the data and unified labels. There were 3.355 registers for which we could not determine the kind of test used and the result obtained, so we removed them from the dataset. The total number of tests was, therefore, 58.635, with 39.191 PCR and 19.444 with rapid antigen tests. On the other hand, the dataset included 6.201 tests from within the Canary Islands, which we did not include in this study, since it focuses on travelers from other territories.

**Figure 1:**
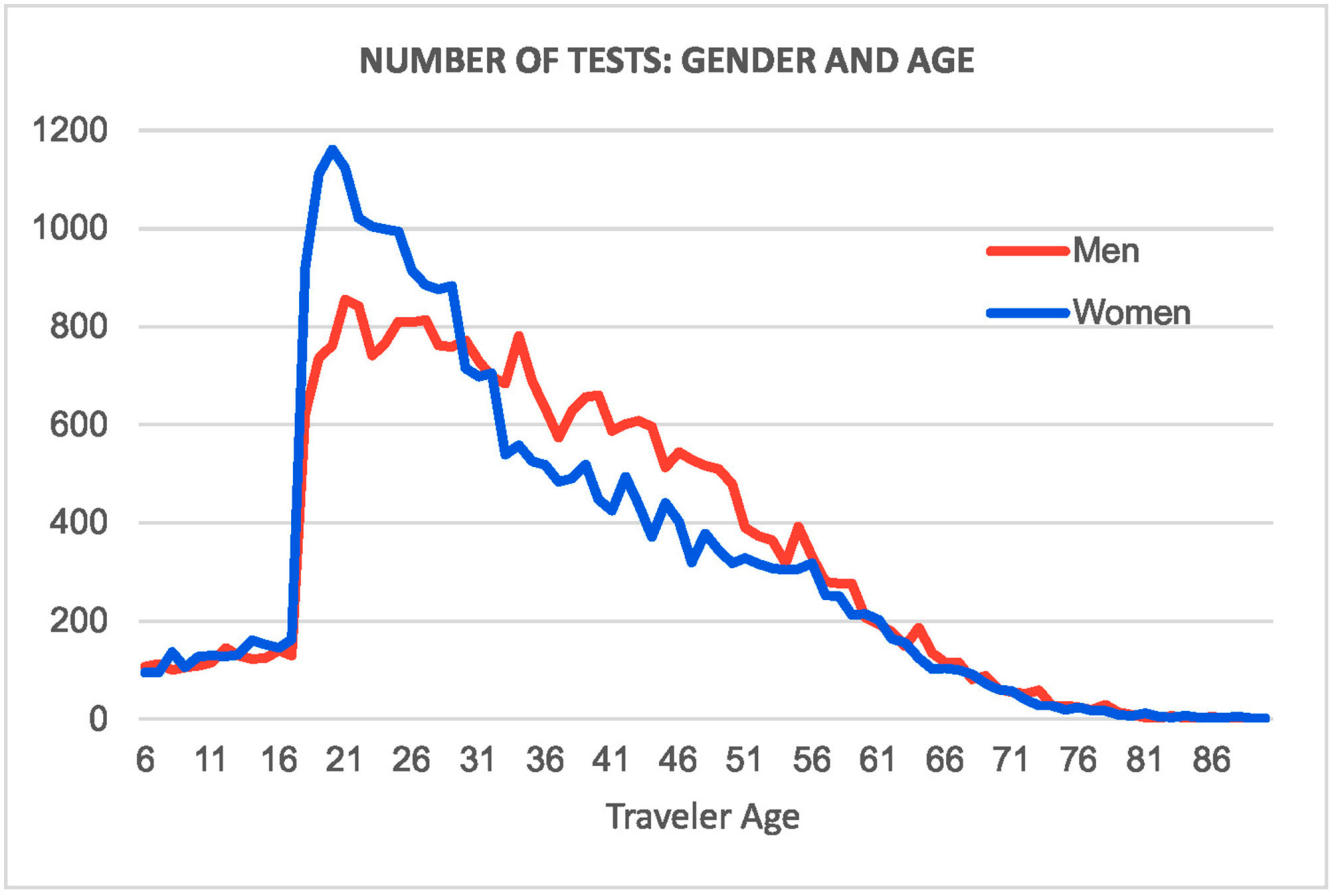
Gender and age of studied travelers.

Additionally, we found 840 duplicated tests, containing both PCR and antigen results. In this case, we considered only the PCR results because these were more reliable. At the end of this process, there were 36.469 PCR and 15.246 antigen tests, making a total of 51.715 registers. In Figure 1, the gender and age of travelers are graphed.

#### Analysis of Tests

The rate of positive PCR and antigen tests was 1,08% (395 cases out of 36.469) and 0,33% (51 cases out of 15.246), respectively. On average, the global rate was 0,86%. This percentage was important for extrapolating the number of positive cases to the cohort of travelers not included in the report, as we will discuss later.

**Figure 2:**
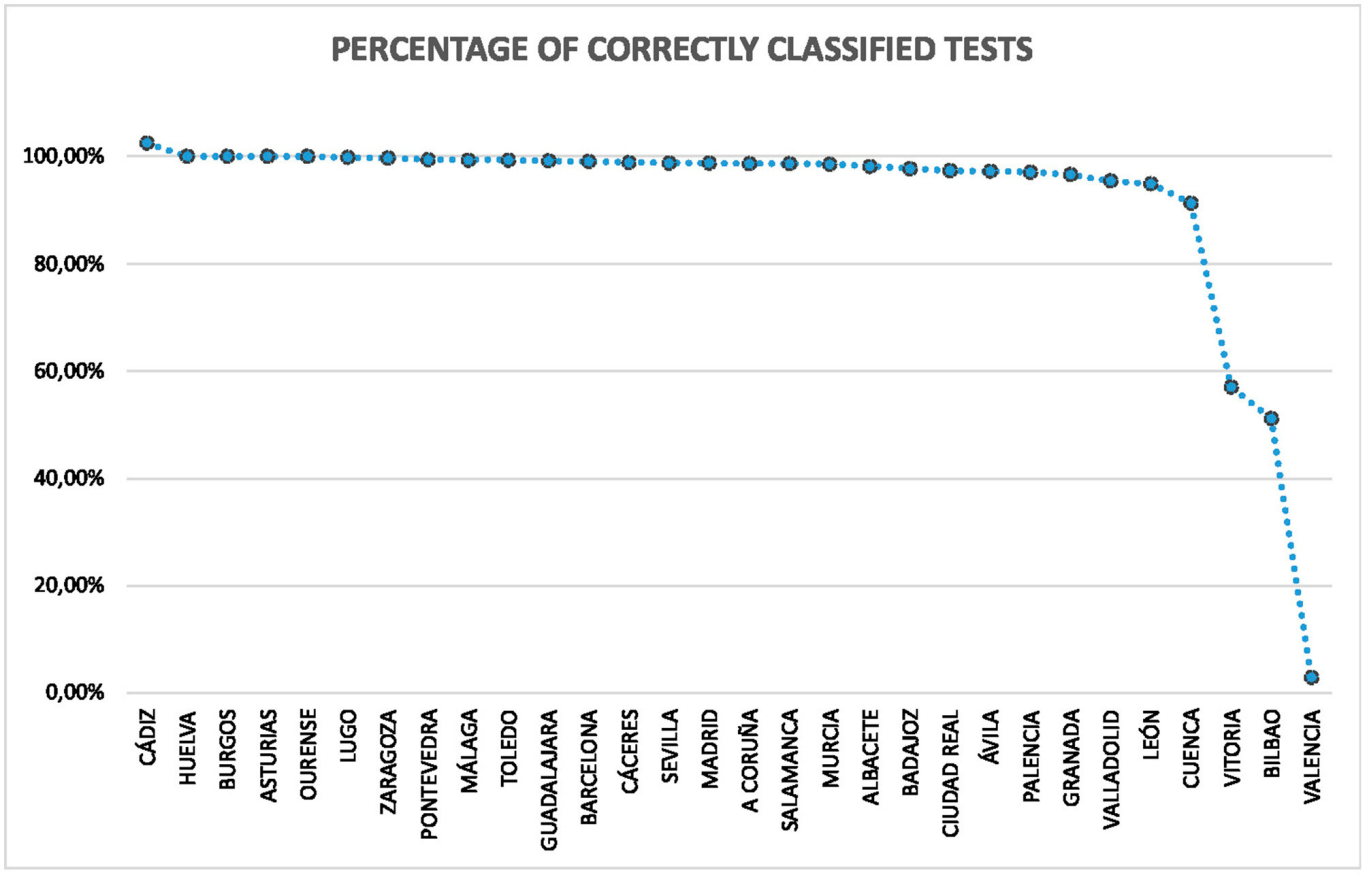
Percentage of correct tests by province.

There were 48 laboratories spread throughout the Spanish provinces. Most of the test results were consistent with a positive or negative classification, with 87,5% of laboratories providing a valid classification for more than 80% of the tests. San Sebastián, Segovia, Vitoria, Bilbao, Valencia, and Palma de Mallorca had a small percentage of valid classifications. In the latter, only four tests were performed, and none obtained a correct result. The Valencia laboratory also obtained a small number of correctly classified tests, with 1.743 tests and only 51 with valid information (2,93% of the total). In the rest of laboratories, the correct number of registers was above 50% of the total number of tests. Figure 2 shows a graph of the percentage of valid tests by province.

**Figure 3:**
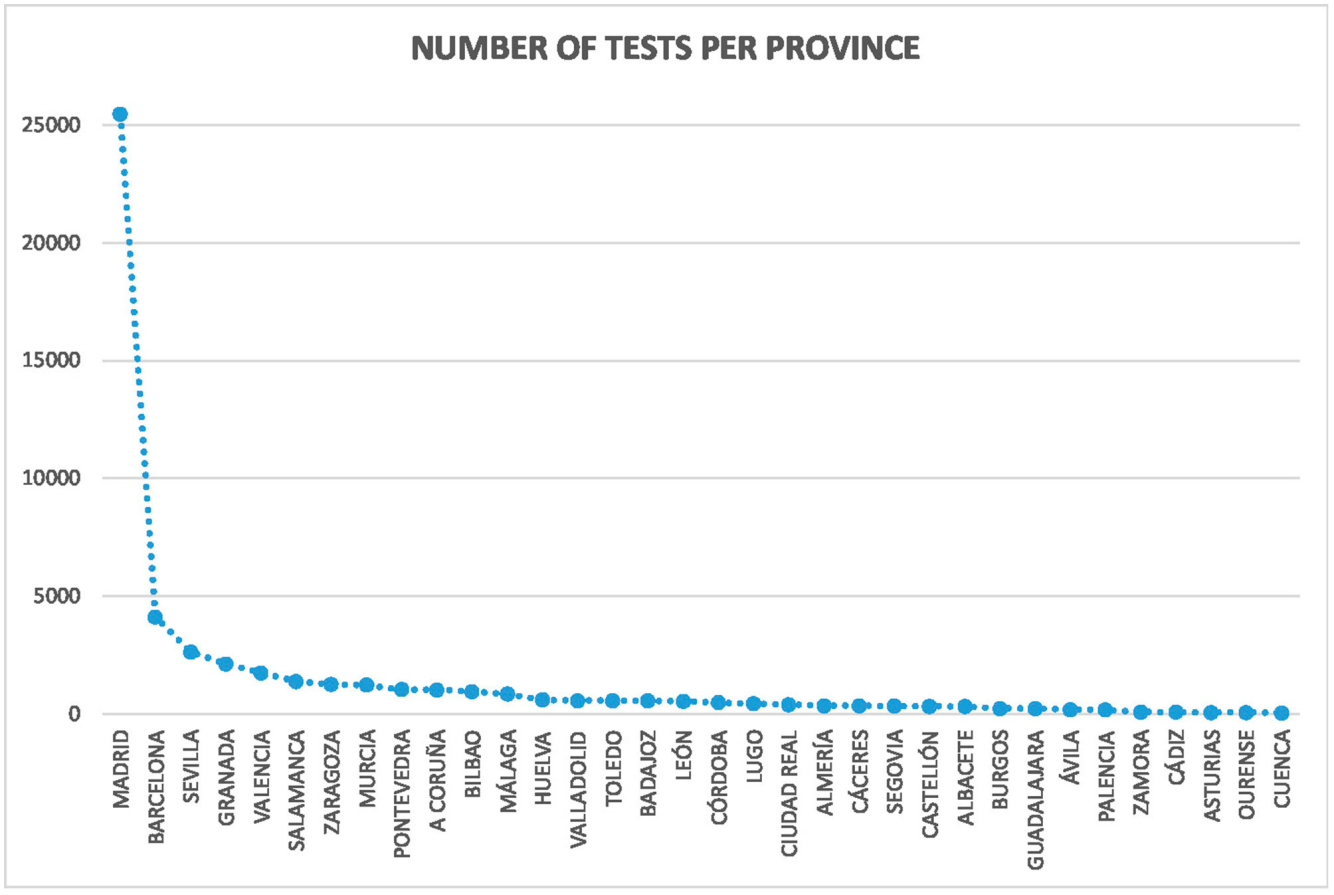
Distributions of tests per province in Spain.

In terms of the number of tests per province, Madrid concentrated the majority with 25.462 in total. This was because its airport served as a transit for flying to the Canary Islands. It is followed by Barcelona (4.119), Seville (2.638), and Granada (2.129). Palma de Mallorca and Menorca were the laboratories with the fewest number of tests (4 in each case). Plasencia was the following locality with 13 tests. The mean number of tests was 1.291,48, with a standard deviation of 3.681,39, and a median of 515,5. On the other hand, the mean number of tests with correct classifications was 1.221,56, with a standard deviation of 3.640,43, and a median of 455. Figure 3 shows the distribution by province.

### Cost-Benefit Analysis Methodology

To assess the cost-benefit of this measure, we first studied the evolution of the COVID-19 pandemic in the Canary Islands. We obtained the number of cases and the rate of hospitalizations during the period between 10 May, 2020 and 28 April, 2020, from the *Instituto de Salud Carlos III* (ISCIII) (7).

On the other hand, the total number of infected travelers could be estimated from the rate of positive tests realized at the laboratories. The number of travelers was provided by Aeropuertos Españoles y Navegación Aérea (AENA), the company responsible for the Spanish airports. Since these groups of people came from the same regions in Spain, we assume that they share the same rate of infections.

This information, together with the rate of hospitalizations obtained from the ISCIII for the Canary Islands, allowed us to estimate the number of incoming persons that would require hospitalization. Additionally, the number of days that a COVID patient spent on average in a Canary Islands hospital was provided by the Government of Canary Islands; therefore, we can estimate the total number of days that the infected travelers would have passed in hospitals.

This was the basis for estimating the costs that the authorities would have covered if no tests were carried out at origin. We calculated the average cost per day for each COVID patient in hospitals, as supplied by the Government of the Canary Islands. This yielded the estimated costs of hospitalizations for the incoming travelers within a confidence interval (CI) of 95%.

On the other hand, we calculated the costs of tests in origin and at the airports from the information provided by the Government of the Canary Islands. The benefit of the testing measures resides, thus, in the difference between the savings of hospitalizations that had been avoided by the tests and the actual costs associated with these tests.

This cost-benefit analysis assumed that no transmission had occurred between the incoming travelers and the local population. Under the hypotheses that no control measures had been carried out, it was more reasonable to think that each infected person would have transmitted the virus to other people. In our first approach, we applied different rates of transmissions to observe the evolution of savings.

We then refine the previous approach by considering the evolution of the pandemic in the Canary Islands. Since we have information about the evolution of the pandemic provided by the ISCIII, we can estimate the increment of transmissions due to the incoming infected persons. In this case, we apply the observed rate of transmissions in the Canary Islands to the incoming travelers. We can obtain a daily estimation of the evolution based on the date of travel of each person and the information of the pandemic. This gives us a conservative evolution of cases if no special measure would have been taken for travelers and the impact on the local population. This study allows us to determine the day from which the measure starts to be beneficial for the administration.

## Results

In this section, we evaluate the cost-benefit of COVID-19 tests in origin. We, first, study the economic impact of positive cases if no controls were established at the airports. We consider the rate of transmissions and the rate of hospitalizations derived from the previous section.

Then, we estimate the total costs from the information of hospitalization expenses. To obtain the economic benefit of the measure we consider the actual costs assumed by the Government of the Canary Islands. We remark here that our study calculates the cost-benefit considering only the information of incoming travelers and, on the other hand, forecasting the evolution of the pandemic if travelers transmit the virus to the local population.

### Evolution of the COVID-19 Pandemic in the Canary Islands

The Canary Islands is a fragmented region composed of eight islands in the northwestern part of the African shore, close to Morocco and Western Sahara. Table 1 shows the total population, which is bigger than 2,000,000 in 2020 and represents 4,73% of the Spanish population. The biggest islands, Tenerife, and Gran Canaria, contain more than 800.000 people each, while the smallest one, La Graciosa, is around seven hundred.

**Table 1:** Population in the Canary Islands and Spain. *Source:* Instituto Nacional de Estadística (INE).

**Region**	**Population in 2020**
Canary Islands	2.244.423
(% with respect to Spain)	4,73
Spain	47.394.223
(including Canary Islands)	

The total number of infections from 10 May, 2020 to 28 April, 2021, is given in Table 2. This information is obtained from the COVID-19 report (7) of the *Red Nacional de Vigilancia Epidemiológica* (RENAVE) belonging to the *Instituto de Salud Carlos III* (ISCIII) and does not include data from the first wave, as it is not reliable.

**Table 2:** Number of positive cases, hospitalizations and ICU admissions in the Canary Islands and Spain (10 May, 2020–28 April, 2021).

**Region**	**Cases**	**Hospitalizations**	**ICU**
Canary Islands (%)	49.969 100	4.197 8,4	870 1,7
Spain (%)	3.271.060 100	238.891 7,3	22.638 0,7

**Figure 4:**
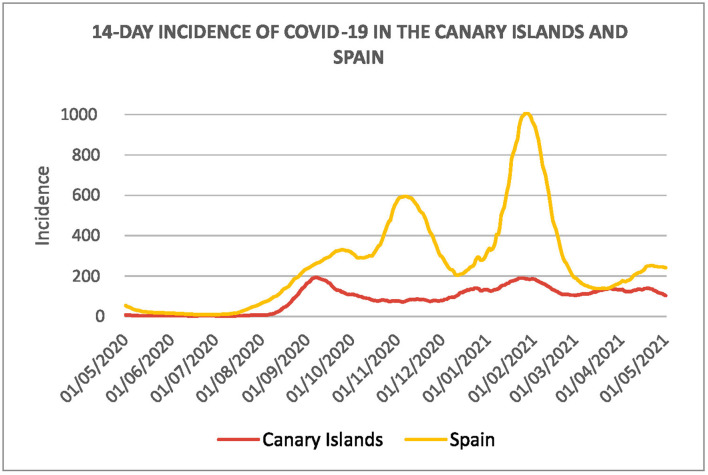
Fourteen days cumulative incidence of the COVID-19 in the Canary Islands and Spain from 10 May, 2020 to 28 April, 2021.

The percentage of hospitalizations and ICU admissions in the islands is about 8,4% and 1,7%, respectively; see Table 2. This information is particularly different from the average of Spanish regions, especially in the case of ICU admissions. The incidence of the COVID-19 pandemic in the Canary Islands and the rest of Spain is also different, as we can see in Figure 4. During this period, two major waves are remarkable in the mainland but less prominent in the case of the islands.

**Figure 5:**
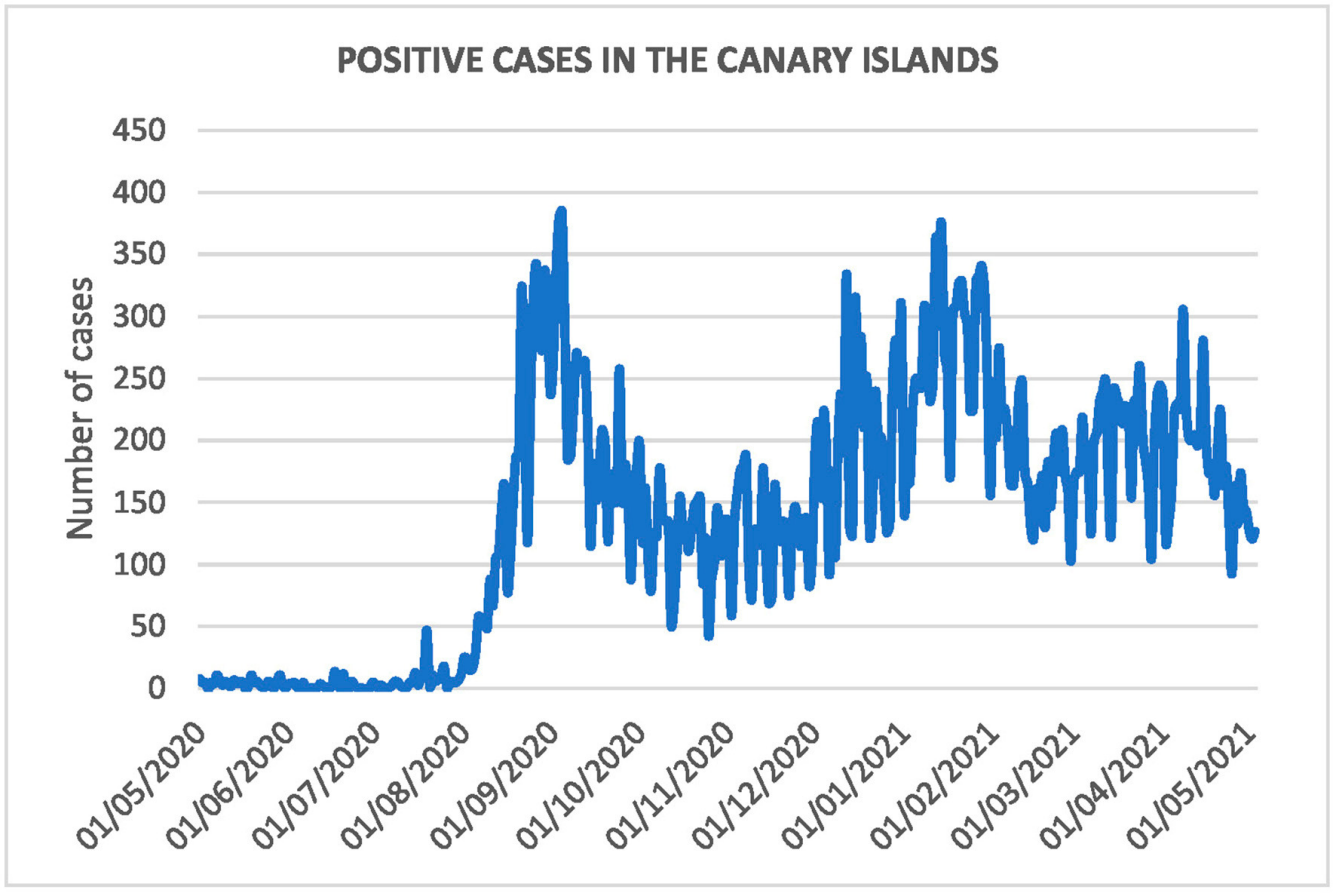
Number of positive cases during the period.

On the other hand, the daily evolution of positive cases, from May 2020 to May 2021, is shown in Figure 5. The number of cases started to increase in August 2020 and declined in October 2020. It continued with a new wave in mid-December 2020, which lasted until February 2021. A slight rebound occurred in March and a final decline at the end of April, 2021. The last wave coincides with our period of study.

### Direct Cost-Benefit Analysis

Table 3 lists the total number of tests done by the laboratories and the number of positive cases detected. We can calculate the rate of positive cases with respect to the total number of tests, which is 0,86%.

**Table 3:** Total number of tests performed by the laboratories and the number of positive cases.

	**Reported tests**	**Non-reported tests**	**Total travelers**
Number of tests	51.715	294.734	346.449
Positive cases	446	2.542	2.988
Positive cases (%)	0,86	0,86	0,86

*Source: Own elaboration, based on data from AENA and estimations*.

The total number of persons that arrived in the Canary Islands airports from abroad in the period of study was 346.449. This allowed us to get the number of people not included in the report that also traveled to the islands. Applying the same rate that we obtained from the tests, we obtained a total of 2.988 positive cases for the whole number of travelers. This assumption was reasonable as we knew that the two cohorts shared the same features as follows: They traveled to the Canary Islands between 14 December, 2020 and 4 April, 2021, and they came from the same regions.

Given the rate of hospitalizations and ICU admissions in the Canary Islands, as given in Table 2 (8,4 and 1,7%, respectively), we can obtain an estimate for our cohort based on the number of positive cases in Table 3. This yields a total of 251 hospitalizations and 52 ICU admissions; see Table 4.

**Table 4:** Estimated number of hospitalizations and ICU admissions.

**Canary Islands**	**Positive cases**	**Positive cases (%)**	**Study cases**	**Estimated cases**	**Total estimation**
Positive cases	49.969	100,00	446	2.542	2.988
Hospitalizations	4.197	8,40	37	213	251
ICU admissions	870	1,74	8	44	52

*Source: Own elaboration*.

The average number of days per patient in hospitals is supplied by the SCS; see Table 5. In the case of hospitalizations, the average number is 13,59 and, in the case of ICUs it is 17,92. This allows us to estimate the occupancy of hospitals. The 95% CI of the time spent by the patients in hospitals is shown in the table.

**Table 5:** Number of days of hospitalizations based on the average number of days per patient in hospitals.

	**Cases**	**Average number of days (95% CI)**	**Total number of days (95% CI)**
Hospitalizations	251	13,59 (13,04–14,13)	3.409,88 (3.273,53–3.546,23)
ICU admissions	52	17,92 (17,20–18,63)	932,20 (895,02–969,38)

*Source: SCS, Government of the Canary Islands*.

The average estimate is 3.409,88 for the total days of hospitalizations and 932,20 of ICUs. These amounts are based on the positive cases detected and the extrapolation to the rest of travelers, and do not include any kind of transmission.

The information of the average cost per day and patient in the Canary Islands hospitals is obtained from the SCS. The hospitalization costs were carried out in nine hospitals. Four “tertiary-level hospitals” in Gran Canaria and Tenerife; three “secondary-level hospitals” in Lanzarote, Fuerteventura; and La Palma and two other minor hospitals in La Gomera and El Hierro where the patients did not demanded ICU in the studied period. According to the OMS definition of this hospital classification: “Secondary-level hospital is highly differentiated by function with 5–10 clinical specialties; size ranges from 200 to 800 beds; often referred to as a provincial hospital.” “Tertiary-level hospital is highly specialized staff and technical equipment—for example, cardiology, ICU, and specialized imaging units; clinical services highly differentiated by function; could have teaching activities; size ranges from 300 to 1.500 beds.”

With this information, and the total number of days given in Table 5, we can calculate the total cost for all the incoming travelers, as shown in Table 6. The expenses in this table provide an estimate of the amount that the Government of the Canary Islands should cover if the incoming travelers were not controlled at the airports.

**Table 6:** Patient hospitalization cost per day, and total cost of estimated positive cases for incoming travelers.

	**Cost per day (€)**	**Total cost (CI) (in €)**
Hospitalizations	273,70	933.280,19 (895.961,18–970.599,19)
ICU admissions	1.985,95	1.851.306,00 (1.777.460,45–1.925.151,54)
		Total cost: 2.784.586,19

*Source: SCS, Government of the Canary Islands*.

On the other hand, each PCR test costs €48,00 and a rapid antigen test costs €24,00. We multiply these quantities by the total number of tests, including the 840 duplications. Another cost is associated with tests performed at the Canary Islands airports for those travelers who arrived without one. The costs of both tests and airport control are given in Table 7.

**Table 7:** Costs of tests and airport control.

**Concept**	**Price/Unit**	**Quantity**	**Total (€)**
PCR tests	48,00 €	36.469	1.750.512,00
Rapid antigen tests	24,00 €	16.086	386.064,00
Airport control			384.922,71
			Total cost: 2.521.498,71

From the information of Tables 6, 7, we calculate the difference between the costs assumed by the Government of the Canary Islands (Table 7) and the savings that this measure produced to the Canary Islands hospitals (Table 6). We conclude that the hospitalization costs would be bigger than the cost of the tests. This means that this measure is beneficial even if only the infected persons traveling from abroad would have required hospital care. The savings are €263.087,47 (95% CI €151.922,92–374.252,02). However, this is not realistic since incoming positive cases will transmit the virus to the local population.

**Figure 6:**
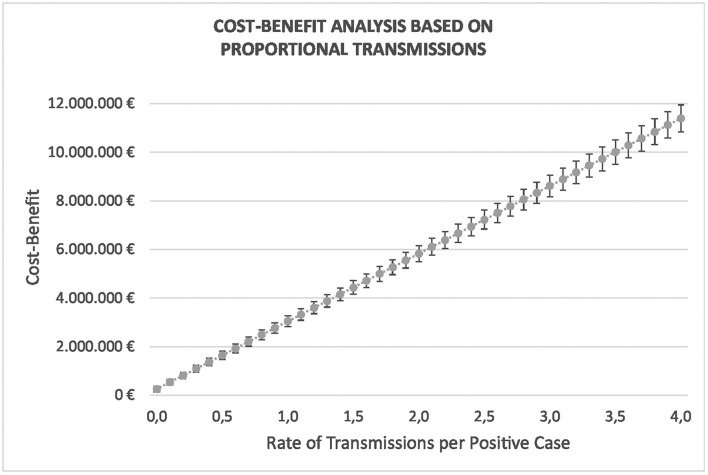
Evolution of the savings when the transmission rate is between 0 and 4, i.e., when every infected traveler transmits the virus to no one else or up to four persons on average. Proportional rate of transmissions is assumed.

If we assume a proportional rate of transmissions, i.e., each positive case transmits the virus to a given number of persons on average, the benefits of the measure would increase, as shown in Figure 6. The first value in the figure (€263.087,47) represents the benefit when there is no transmission at all (rate 0), as discussed above. A 0,5 rate of transmission means that every two positive cases infected another person, and a rate of four means that every infected traveler transmits the virus to four persons.

The evolution of the graph reveals the economic benefit of the measure with respect to a proportional rate of transmissions. This illustrates the important savings that can be obtained. In the next section, we make a more realistic forecast of the transmissions according to the actual evolution of the pandemic.

### Cost-Benefit Analysis of Estimated Transmissions

It is interesting to study the impact during the evolution of the pandemic. In this section, we forecast the propagation of the virus caused by the incoming travelers and study the daily evolution of positive cases. This will allow us to estimate the daily impact and, additionally, it will permit us to estimate the date from which this measure starts to be profitable.

We first performed an analysis of the test data for Canary Islands residents supplied by the laboratories to obtain the rate of positive cases. Then we extended this rate to the total amount of travelers during the period. This yielded an estimate of the total number of infected persons. Then, we have calculated the ratio of hospitalizations and ICU admissions in the Canary Islands, which was 8,4 and 1,74%, respectively. Applying these rates to the infected travelers allowed us to obtain an estimate of the minimum number of persons that would need medical care in hospitals. This approach was conservative since it represented a low boundary in the number of possible new infections. It was reasonable to think that the real impact would be slightly bigger than in our study because the basic reproduction number might have increased. In this case, hospitalization costs would be higher, and the benefits of PCR or antigen tests in origin would be still more favorable.

**Figure 7:**
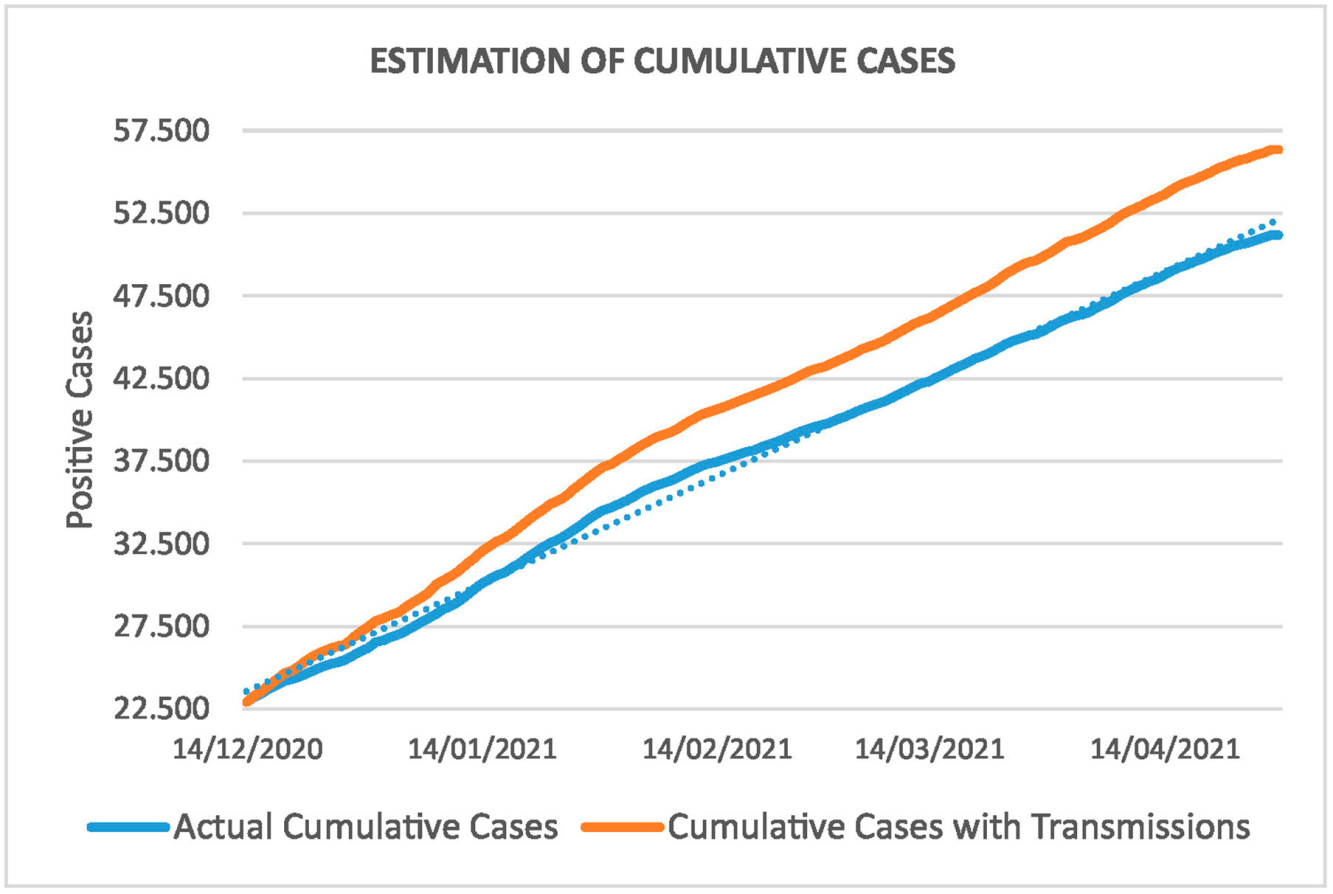
Cumulative cases in the Canary Islands. The blue line represents the actual evolution of the pandemic and the orange one shows the forecasting if we consider transmissions from infected travelers.

Figure 7 shows the cumulative cases in the Canary Islands from 14 December, 2020 till the end of April, 2021. The blue line is the data evolution as published by the ISCIII (7). We observe that the pandemic has evolved linearly, starting from 22.916 positive cases to 46.532 and 51.205 on the 4 and 28 of April 2021, respectively.

The orange line in Figure 7 is the forecasting of transmissions of the virus if travelers are not controlled at-origin. We assume that the incoming travelers suffer the same linear evolution as the actual curve. As the information is registered daily, we can simulate the increment by applying the same percentage of increment of the cumulative actual curve. In this case, the 446 positive cases would become 699,56 on April 4, and 769,81 on April 28.

For the rest of travelers, we distribute the 2.542 positive cases according to the distribution of the data during the 4 months period. This is reasonable since both populations have the same features and we should expect a similar behavior; 42,2% of travelers who entered the Canary Islands on domestic flights, between December 2020 and April 2021, were residents (8). Thus, the total number of positive cases, 2.988, as given Table 3, becomes 4.686,47 and 5.157,11 infected persons on April 4 and 28, respectively.

**Figure 8:**
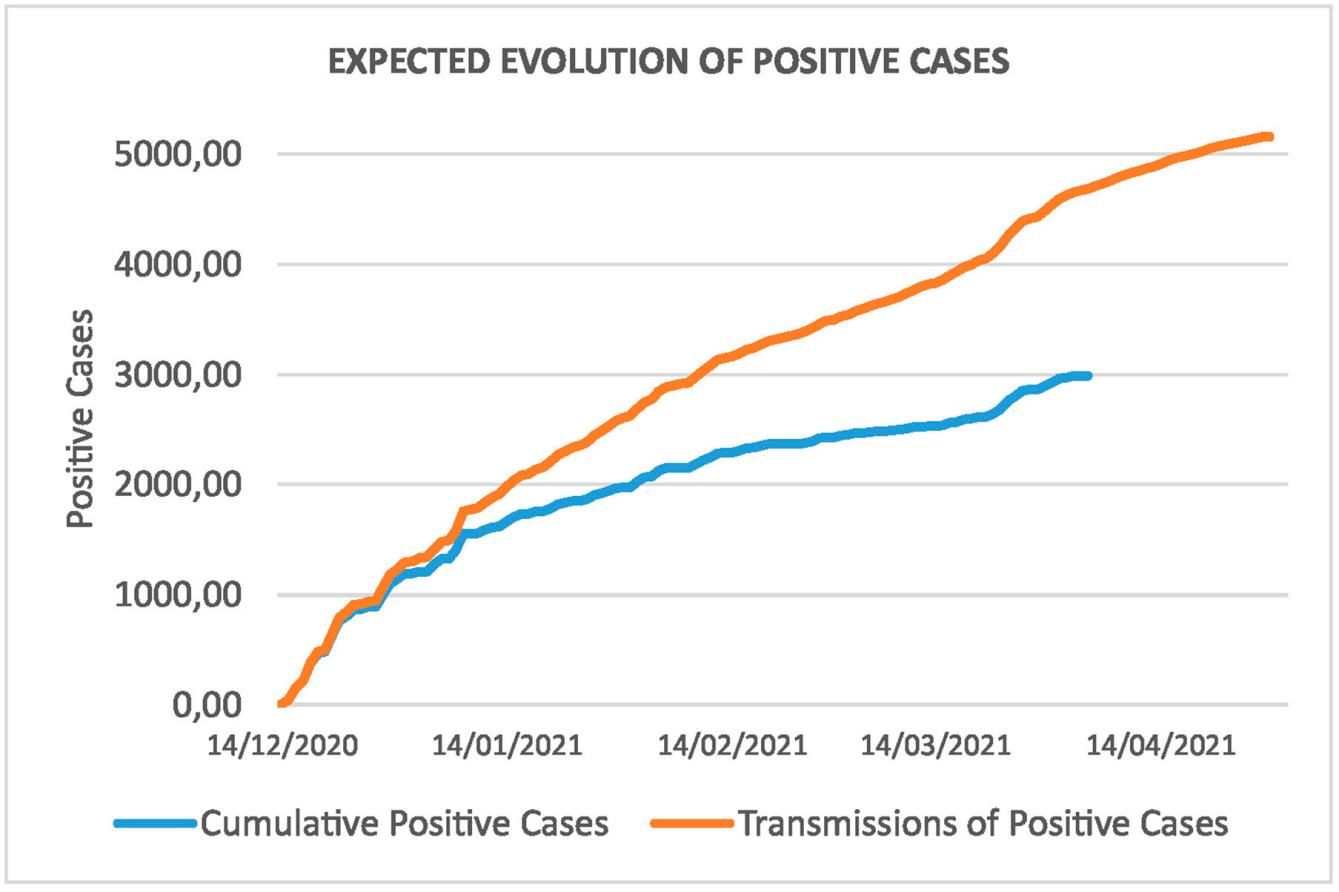
Cumulative cases of newcomers and estimated transmissions.

Looking at the orange curve, this amounts to a total of 51.218,47 and 56.362,11 on both dates. According to the rate of hospitalizations in Table 4, this produces a total of 393,63 hospitalizations and 81,60 ICUs on April 4, and 433,16 and 89,79 on April 28, respectively. Note that we have an estimation of the daily evolution in both cases. Figure 8 shows this evolution for the total number of positive cases of the incoming travelers. The blue curve stands for the real cases and extrapolates for the rest of travelers, and the orange curve forecasts the evolution following the same distribution as the curve given by the ISCIII (7).

**Figure 9:**
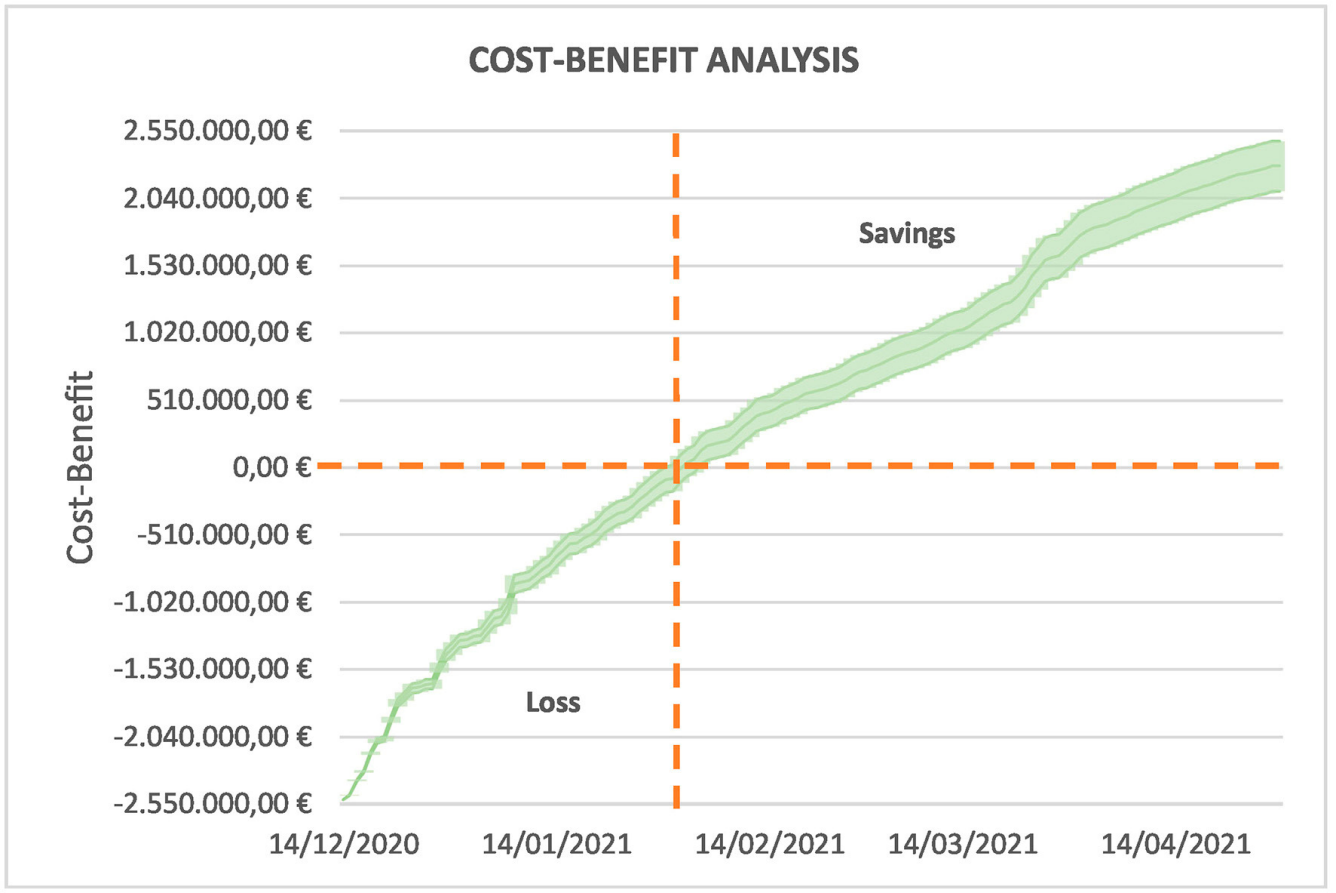
Daily evolution of savings for the public budgets. The measure starts to be beneficial from the beginning of February and the savings exceed €2.000.000 by the end of April.

Using the information from Tables 5–7, it is easy to derive the economic impact of the measure in detail. Figure 9 depicts the evolution of the cost-benefit analysis during the period. Assuming that expenses are accounted for at the beginning of the period, this graph shows the evolution of losses or savings according to the estimated number of hospitalizations. This allows us to determine the day when this policy starts to be beneficial.

At the beginning of February (2 February), the curve becomes positive, and the savings are around €130.551,47, with a 95% CI of €24.677,94 – 236.425,00. This is just 1 month and a half after the beginning. At the end of April, the savings are above €2.000.000 (€2.284.788,50 on average and 95% CI of €2.092.914,84 – 2.476.662,16) and, if the pandemic continues, savings will also increase.

## Discussion

The World Health Organization (WHO) (6) has promoted preventive measures for reducing the impact of the pandemic. One of these measures was tests in origin for travelers. Given the complexity of the task, it is difficult, as pointed out in (9) to provide undisputable evidence for or against the effectivity, efficacy, and effectiveness of many current interventions, included the tests. In this work, we have analyzed the results of this strategy in the scope of the Canary Islands during a period of 4 months, with a focus on the economic impact for the Regional Government. Our study is conservative from an economical point of view since it is based on mild assumptions about the spread of the virus from the incoming travelers.

In a first step, we filtered and unified the test data supplied by the company. The classification of positive and negative tests was not uniform, and we corrected many details. We also removed registers for which it was not clear the classification and we only considered the cost of the PCR test for duplicated items, because it was more expensive and reliable. The rate of positive cases with respect to the total number of tests is 0,86%. In (9), it is quantified unpublished data on PCR screening of departing travelers from South Africa for the period March to November 2020 in <1% [6 of 833 screened passengers (0.72%)]. A simulation study (4) identified 0,6% travelers would be actively infectious on the day of travel. Other authors (2) identified difficulties in proper accounting due in part to the wide divergence of measures taken by airlines, by countries and by regions within each country. Similar studies (10) found a low percentage (0,05%) of airline passengers identified with active SARS-CoV-2 infection on rapid antigen testing during travel from the USA to Italy.

The information from the laboratories of the company was reliable in general. Only six laboratories had low rates of correct classifications and were carefully filtered out. Most tests came from a single province (Madrid). This was because its airport served as a hub for flying to the Canary Islands and many people moved first to Madrid to take the plane, passing the test in the laboratory of this province. This did not affect our analysis because it was based on the total number of transmissions and not on the origin of each test.

The evolution of the pandemic in the islands was different from the rest of the national territory and the behavior in each island was also different in general. In this study, we considered the global evolution of the positive cases in the archipelago, as the incoming traveler data did not include information about the destination airport, and we could not particularize it for each island. Therefore, we assumed that the distribution of the travelers in the islands should correlate with the COVID-19 incidence in the whole region; however, we had no means to check this relation. In any case, the relevance of the two most populated islands in the evolution of the pandemic was quite remarkable, and both receive a similar number of travelers every year, so we might expect that the distribution of newcomers might be aligned with our estimates.

The period of study coincides with a large wave of COVID-19 incidence, which is much more intense in the rest of the country. This means that if no control had been carried out at the airports of origin, many more positive cases than those detected by the tests would have entered in the islands, and the situation would have been worse. Without cost-benefit analysis (4) their findings support adoption of testing strategies for COVID-19 to reduce the risk of infection from travel.

The information about the average number of days that a patient spends in the hospital and the cost per day allow us to estimate the total cost of hospitalizations. This information is obtained from the hospitals and averaged according to the number of beds in each one. On the other hand, we calculated the costs of the tests and those realized at the Canary Islands airports. Comparing these amounts, we observe that the measure is profitable even if only the infected travelers would have been treated at hospitals.

We then studied the propagation of the COVID-19 virus due to these incoming people. We assumed the evolution to be like propagation in the Canary Islands, according to the information provided by the ISCIII. The investment in antigen tests and airport controls yields important savings as soon as 1 month and a half after the beginning of the measure when total expenses are countervailed by savings in hospitalizations costs. At the end of the period, the savings are double the expenses.

In this study, we did not include the number of deceases due to COVID-19 or the epidemiological risk in the region (11). However, we can easily calculate the saving of human lives. According to (12), the percentage of deceases was 1.1% on April 7 in the Canary Islands. As we have 4.686,47 and 5.157,11 infected persons on April 4 and 28, the estimated deceases are about 52 and 57 persons, respectively. Even if it is difficult to calculate the economic cost of deaths, we can rely on the value of a statistical life (VSL), which is a measure of mortality risk reduction and is typically used for cost-benefit analyses. In the recent work by Viscusi (13), the author lists the VSL of 100 countries during the COVID-19 pandemic. The estimate for Spain is $6,67 million per person, or 5,77 million euros. This means that the mortality cost is about 300,04 and 328,89 million euros for the expected deceases. The impact of travel-related control measures for containment of the SARS-CoV-2 pandemic is summarize (14) in a rapid evidence map but no cost-effectiveness analysis is included.

## Conclusions

Preventive measures have been important for restraining the spread of the COVID-19. Tests in origin are especially a good measure that have helped to mitigate the spread among regions.

We study the region of Canary Islands, the Spanish ultra-peripheric archipelago, by studying 61.990 reported data during 2020 and 2021 from travelers from national flights, against 346.449 of total incoming travelers to Canary Islands in this period.

Our results confirm that the saving produced to the public budget have been quite relevant and representative. The measure pursued by the Government of Canary Islands of providing free PCR or rapid antigen tests for residents shows a clear benefit for both, limiting the propagation of COVID-19 and reducing the costs of hospitalizations and ICU admissions. It should be noted that the free testing measure in this period was before starting the vaccination campaigns. As a measure of public health in airports, testing helped to control and make secure the mobility of travelers. Fortunately, the focus at present moment is the massive vaccination developed in Spain to population. Although the vaccination has fulfilled the expectative and it has been progressively dominant as the preferred preventive measure, still PCR and rapid antigen test can be considered as a secure method for some residual cases where vaccination is not completely available.

The incidence in this region has been typically smaller than in the mainland, so the effect of not controlling incoming travelers would have caused an important increased in the hospitalization costs.

While this study is not sufficient to assess the whole savings produced to the public budget, it outlines an effective measure where citizens and governments serve as a powerful strength to mitigate the pandemic. In future research, an evidence synthesis may be accomplished to evaluate not only travel-related control measures but some other strategies addressed by the public health system.

## Data Availability Statement

The original contributions presented in the study are included in the article/supplementary material, further inquiries can be directed to the corresponding author.

## Author Contributions

CD and RG: conceptualization, resources, supervision, project administration, and funding acquisition. CD, RG, JSá, and JSu: methodology. CD, RG, JSá, JSu, and CD-Q: investigation, original draft preparation, and review and editing. JSá: data curation. JSá and JSu: visualization. All authors have read and agreed to the published version of the manuscript.

## Conflict of Interest

The authors declare that the research was conducted in the absence of any commercial or financial relationships that could be construed as a potential conflict of interest.

## Publisher's Note

All claims expressed in this article are solely those of the authors and do not necessarily represent those of their affiliated organizations, or those of the publisher, the editors and the reviewers. Any product that may be evaluated in this article, or claim that may be made by its manufacturer, is not guaranteed or endorsed by the publisher.
